# High-Resolution and Non-destructive Evaluation of the Spatial Distribution of Nitrate and Its Dynamics in Spinach (*Spinacia oleracea* L.) Leaves by Near-Infrared Hyperspectral Imaging

**DOI:** 10.3389/fpls.2017.01937

**Published:** 2017-11-09

**Authors:** Hao-Yu Yang, Tetsuya Inagaki, Te Ma, Satoru Tsuchikawa

**Affiliations:** ^1^Northeast Institute of Geography and Agroecology, Chinese Academy of Sciences, Changchun, China; ^2^Graduate School of Bioagricultural Sciences, Nagoya University, Nagoya, Japan

**Keywords:** nitrate content, near-infrared hyperspectral imaging, partial least squares, vegetable

## Abstract

Nitrate is an important component of the nitrogen cycle and is therefore present in all plants. However, excessive nitrogen fertilization results in a high nitrate content in vegetables, which is unhealthy for humans. Understanding the spatial distribution of nitrate in leaves is beneficial for improving nitrogen assimilation efficiency and reducing its content in vegetables. In this study, near-infrared (NIR) hyperspectral imaging was used for the non-destructive and effective evaluation of nitrate content in spinach (*Spinacia oleracea* L.) leaves. Leaf samples with different nitrate contents were collected under various fertilization conditions, and reference data were obtained using reflectometer apparatus RQflex 10. Partial least squares regression analysis revealed that there was a high correlation between the reference data and NIR spectra (*r*^2^ = 0.74, root mean squared error of cross-validation = 710.16 mg/kg). Furthermore, the nitrate content in spinach leaves was successfully mapped at a high spatial resolution, clearly displaying its distribution in the petiole, vein, and blade. Finally, the mapping results demonstrated dynamic changes in the nitrate content in intact leaf samples under different storage conditions, showing the value of this non-destructive tool for future analyses of the nitrate content in vegetables.

## Introduction

Nitrate is a salt of nitric acid that occurs naturally in the environment. It is found in the soil and is taken up by all plants to be used as a primary nitrogen source. However, high levels of nitrate fertilization also contribute to elevated nitrate contents in the harvested plants. In particular, leafy vegetables that are grown in the soil accumulate high concentrations of nitrates in their leaves and stems, which are then consumed by humans as a rich source of inorganic nitrate. In the human body, nitrate can be converted into nitrite by microbial reduction, which can lead to methemoglobinemia and the formation of N-nitroso compounds, which are carcinogenic (Böhmer et al., 2016). Many countries have set regulations to control the maximum levels of nitrate in foodstuffs, and consequently, a reduction in the nitrate concentration in vegetables has been considered at production sites.

To effectively reduce the nitrate content in vegetables, understanding of nitrate metabolism is essential. Nitrate is absorbed through the plant roots and reduced to nitrite by nitrate reductase (NR) in the cytoplasm. This reduction reaction is a rate-limiting step in the nitrate assimilation pathway in higher plants (Forde, 2000; Chen et al., 2004). Therefore, although nitrate accumulation can be reduced by some horticultural methods, enhancing NR activation is considered fundamental for decreasing nitrate accumulation. *In vivo* studies on the dynamic changes in nitrate content in leaves are important for analyzing the absorption and transformation of nitrate in plants, which will contribute to improving nitrate use efficiency, thereby reducing its accumulation. However, the traditional method for determining nitrate content requires samples to be pulverized, which makes it difficult to obtain consecutive readings (Itoh et al., 2011). Therefore, the establishment of a rapid, non-destructive method for detecting nitrate is important for monitoring and controlling vegetable nitrate content.

Near-infrared (NIR) spectroscopy is currently recognized as one of the most powerful, rapid, and non-destructive techniques for measuring the material composition and internal qualities of fruits and vegetables (Tallada et al., 2006; Ecarnot et al., 2013; de Oliveira et al., 2014). This technique detects absorption in the NIR region (780–2,500 nm), which arises from the vibrations of C-H, O-H, and N-H groups, and has been successfully used to measure nitrate content. For example, Ito et al. (2003a) detected the visible-NIR absorption spectra of Japanese radish (*Raphanus sativus*) rhizomes and used stepwise regression to establish a calibration model using four wavelengths. Following this, Ito and Idezawa (2006) developed a multiple linear regression equation using four wavelengths to enable the nitrate content in the leafy vegetable Qing gin cai (*Brassica chinensis*) to be determined non-destructively, and demonstrated that the nitrate spectral characteristics at 514 nm were not affected by the chlorophyll and carotenoid contents of the vegetables. In addition, Xue and Yang (2009) developed models to measure the nitrate content in spinach (*Spinacia oleracea*) leaves using the reflection spectrum.

However, conventional NIR spectrometry is based on point measurements and thus it cannot resolve the spatial distributions of constituents within a sample. Near-infrared hyperspectral imaging has been applied to plant and food quality testing and material composition analysis (Diezma et al., 2013; Schmilovitch et al., 2014; Yu et al., 2014). Consequently, spectral imaging is preferred over conventional NIR spectrometry for the evaluation of non-uniform nitrate contents in leaves. NIR hyperspectral imaging has emerged as a new technology for evaluating the composition and quality of vegetables (Higa et al., 2013; Lopez-Maestresalas et al., 2016). This tool can provide an NIR spectral image at each wavelength instead of at just one value, thus enabling quality evaluation across the entire surface. It has emerged as a new technology for food quality evaluation (Itoh et al., 2010, 2011).

The purpose of this study was to evaluate the potential of NIR hyperspectral imaging for determining the distribution of nitrate content in spinach leaves and for mapping dynamic variations in the distribution of nitrate under different storage temperatures.

## Materials and methods

### Sample preparation for calibration

**Figure 1:**
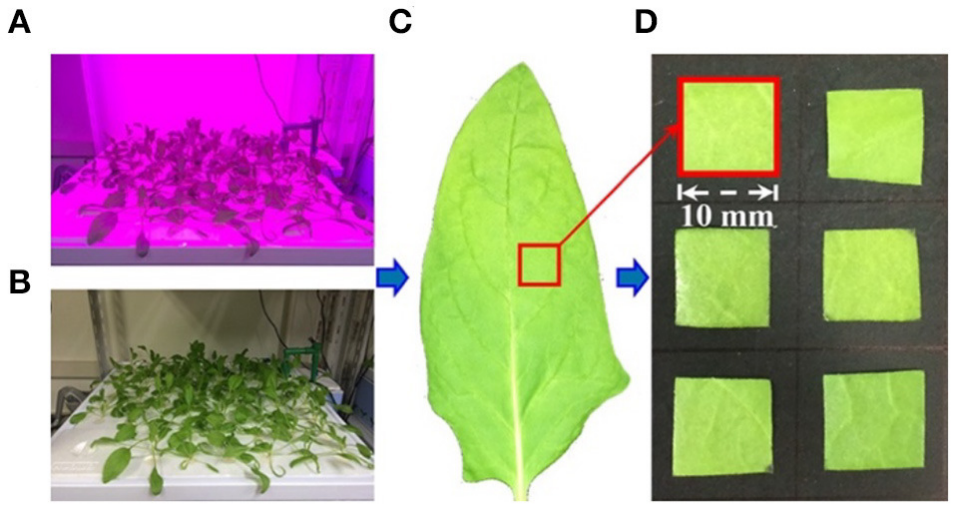
Step of sample preparation. **(A)** Lighting time, 10 h; **(B)** dark time, 14 h; **(C)** calibration sample selection, leaf center position near the main vein; **(D)** calibration sample size, almost 1 cm^2^.

**Figure 2:**
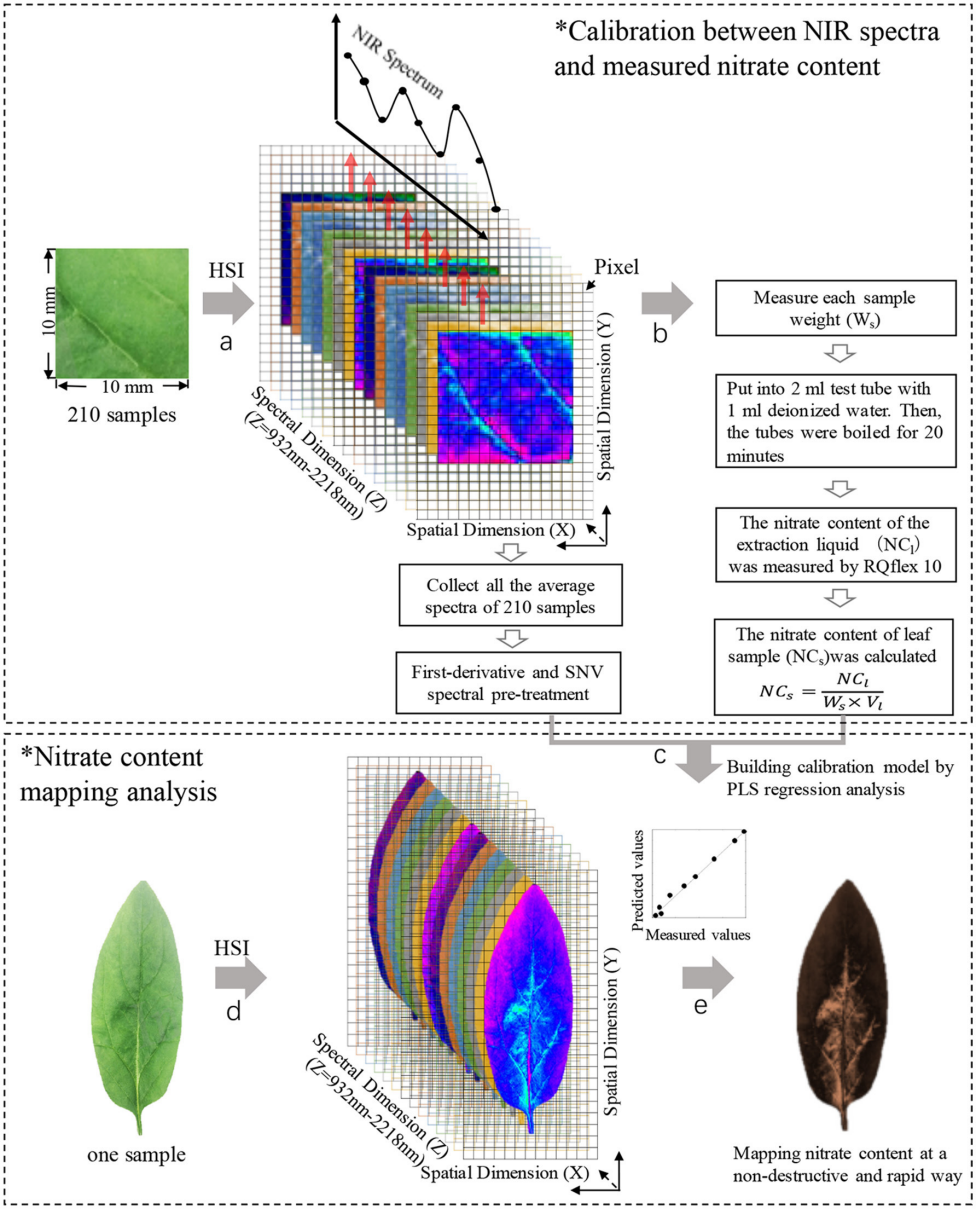
The steps of nitrate content mapping analysis. Step a, collecting the calibration NIR spectrum of samples; step b, measuring nitrate content of samples; step c, building calibration model; step d, collecting NIR spectrum of intact leaf; step e, mapping nitrate content distribution in leaves. HSI, hyperspectral image; SNV, standard normal variate.

The sample preparation steps are shown in Figure 1. Spinach (*Spinacia oleracea* L.) plants were cultivated using a mixture of fertilizer and hydroponic treatments to provide a broad range of nitrate contents. Three concentrations of Hyponika liquid fertilizer (Kyowa Co., LTD., Japan) were used (1.0, 2.0, and 4.0 mL/L). The plants were grown in an artificial light environment established by a blue (20%) and red (80%) light-emitting diode plant tube (LK031; Japan), with 10 h of light per day for each treatment. Seven leaves were collected from each fertilizer condition every 3 d, each collection was started 2 h after turning on the lights. The nitrate contents of samples were measured immediately after the acquisition of near infrared hyperspectral images (Figure 2).

Since the petiole and leaf vein have higher nitrate contents than the leaf blade (Itoh et al., 2010); to increase the likelihood of accurately calculating the nitrate content in a uniform sample, a 1 cm^2^ sample was cut from the center of the leaf avoiding the main vein. A total of 210 samples collected over 10 sampling occasions were measured in this study. Then, the average spectra calculated from all pixels (156 × 156 μm/pixel) in each sample region was used for making a calibration model with the nitrate contents values.

### NIR hyperspectral imaging system

The NIR hyperspectral imaging system consisted of a camera, light source, and sample stage (Compovision™; Sumitomo Electric Industries, Ltd.). The camera possessed a spectroscope and a two-dimensional photosensitive element [256 pixels (wavelength) × 320 pixels (position)], which could receive NIR light in the range of 913–2,519 nm, and the spectrograph had a wavelength step of 6.2 nm. A single line halogen lamp was used as the light source, which was parallel to the line scan detector of the camera and irradiated the sample at 45°. The sample stage moved in a perpendicular direction to the line scan detector and the NIR images were obtained from the surface of each sample using a push-broom line scanning system. The distance between the target and the camera was manually adjusted to achieve a horizontal field of view at 50 mm (one pixel could cover 156 × 156 μm^2^ of the target).

**Figure 3:**
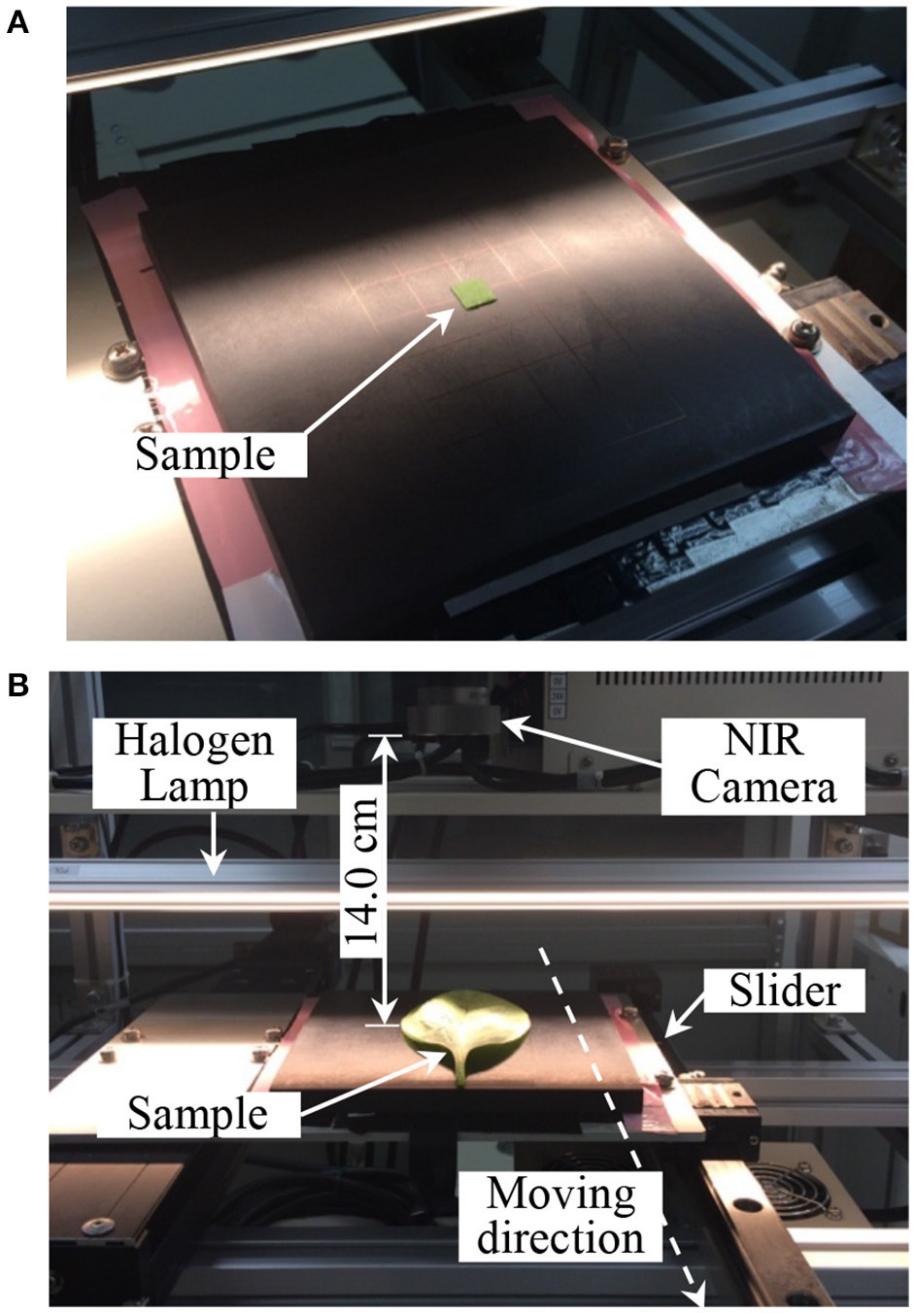
NIR hyperspectral images measurement. **(A)** Calibration HIS data measurement, **(B)** mapping HSI data measurement. HSI, hyperspectral image.

To obtain the spectral images, each sample was positioned on the stage and scanned line-by-line (Figure 3) using an exposure time of 1/150 s and a sample stage movement speed of 23.4 mm/s. As a reference, a white plate was scanned under the same conditions and a dark image was obtained by turning off the light source and completely covering the lens with its cap. Each spectral image of the leaf sample was converted into a relative reflectance value for further analysis using the following equation (Tallada et al., 2006):

(1) $$R_{\lambda ,\;n}=\frac{S_{\lambda ,\;n}-B_{\lambda ,\;n}}{W_{\lambda ,\;n}-B_{\lambda ,\;n}}$$

where *R*_λ*n*_ is the relative reflectance at wavelength λ of pixel *n, S*_λ*n*_ is the intensity of the sample at wavelength λ of pixel *n*, and *W*_λ*n*_ and *B*_λ*n*_ are the intensities of the reference plane and the dark image, respectively, at wavelength λ of pixel *n*.

The NIR hyperspectral imaging system used in this study was push broom method scanning. In this study, a longer exposure time 1/150 s for each image (maximum speed could be 1/320 s) was used to enable a good Signal to Noise Ratio (S/N). Notwithstanding, if the sensitivity among camera pixels varies over time, and differs from the current calibration set, non-periodic stripes become visually perceptible (Rogass et al., 2014). Therefore, we applied the 2D-discrete Fourier transform method which efficiently reduced the stripes noise of sample images (Barigye and Freitas, 2015). The reduction technique can effectively remove the striping, and the calibration model can be improved such that the prediction error becomes smaller.

### Measurement of nitrate content

Immediately after the spectral imaging, the square leaf sample was weighed, placed into a 2-mL test tube with 1 mL deionized water, and boiled for 20 min. Inside the test tube is a closed system, the water vapor will not be lost. The nitrate concentration was measured after the test tube was cooled to ambient temperature (20°C) (Itoh et al., 2010). The nitrate concentration in the extraction liquid was then measured using a RQflex® 10 reflectometer (MERCK, Germany). RQflex measurement was a fast and accurate measuring instrument for liquid samples. Researchers have compared the RQflex® 10 with Ion Chromatography method for detecting the nitrate content. The results indicated that the correlation coefficient between the two methods was 0.996 and the RQflex® 10 was found to be more accurate (Masako and Tadakatsu, 1995). At the same time, Ito (Ito et al., 2003b) also compared the test results of RQflex® 10 with High Performance Liquid Chromatography (HPlC), and almost the same accuracy was achieved. Reflectometric method with nitrate test strips was used, at a measurement range from 5 to 225 mg/l ${\text{NO}}_{3}^{-}$ and measurement accuracy was 1 mg/l. It was used for accurate measuring nitrate content in production of agricultural products and food safety (Rodrigues et al., 2005; Thompson et al., 2009; Rytel, 2012a,b). The nitrate content was calculated according to the following equation:

(2) $$NC_{s}=\frac{NC_{l}}{W_{s}\times \;V_{l}}$$

where *NC*_*s*_ is the nitrate content of the leaf sample, *NC*_*l*_ is the nitrate concentration in the extraction liquid, *W*_*s*_ is the weight of the leaf sample, and *V*_*l*_ is the volume of the extraction liquid.

### Production of a calibration model

Partial least squares (PLS) regression is a recent technique that generalizes and combines the features of principal component analysis and multiple regression. It is particularly useful when a set of dependent variables needs to be predicted from a large set of independent variables. PLS regression is a proven multivariate calibration method for quantitative analysis that overcomes problems concerning overlapping bands and collinearity of data. The main idea is to calculate principal components of the NIR spectra and measured values separately, then to develop a regression model between them (Barboza and Poppi, 2003, refer to Supplemental Data 1). In this study, the NIR hyperspectral image of each leaf sample was obtained by the hyperspectral image system and the average spectrum of each cut sample (1 cm^2^) was calculated. The sample nitrate contents calculated using RQflex 10 were used as reference values to establish prediction models with the averaged NIR spectra by PLS regression. The reference sample data that were used to establish the PLS calibration model are shown in Table 1. The nitrate content of the leaf samples ranged from 2,187 to 8,767 mg/kg, with a mean of 4,852.96 mg/kg. The leaf samples between 3,500 and 5,000 mg/kg were prevalent in our study, such that their overall distribution was shifted slightly toward low nitrate content (Supplemental Data 2).

**Table 1:** Summary of reference values for PLS calibrations.

**Property**	**No**.	**Min**	**Max**	**Mean**	**Median**	***SD***
Nitrate content (mg/kg)	210	2,178.00	8,767.00	4,852.96	4,557.00	1,460.72

*No., number of data point; Min, minimum; Max, maximum; SD, standard deviation*.

**Figure 4:**
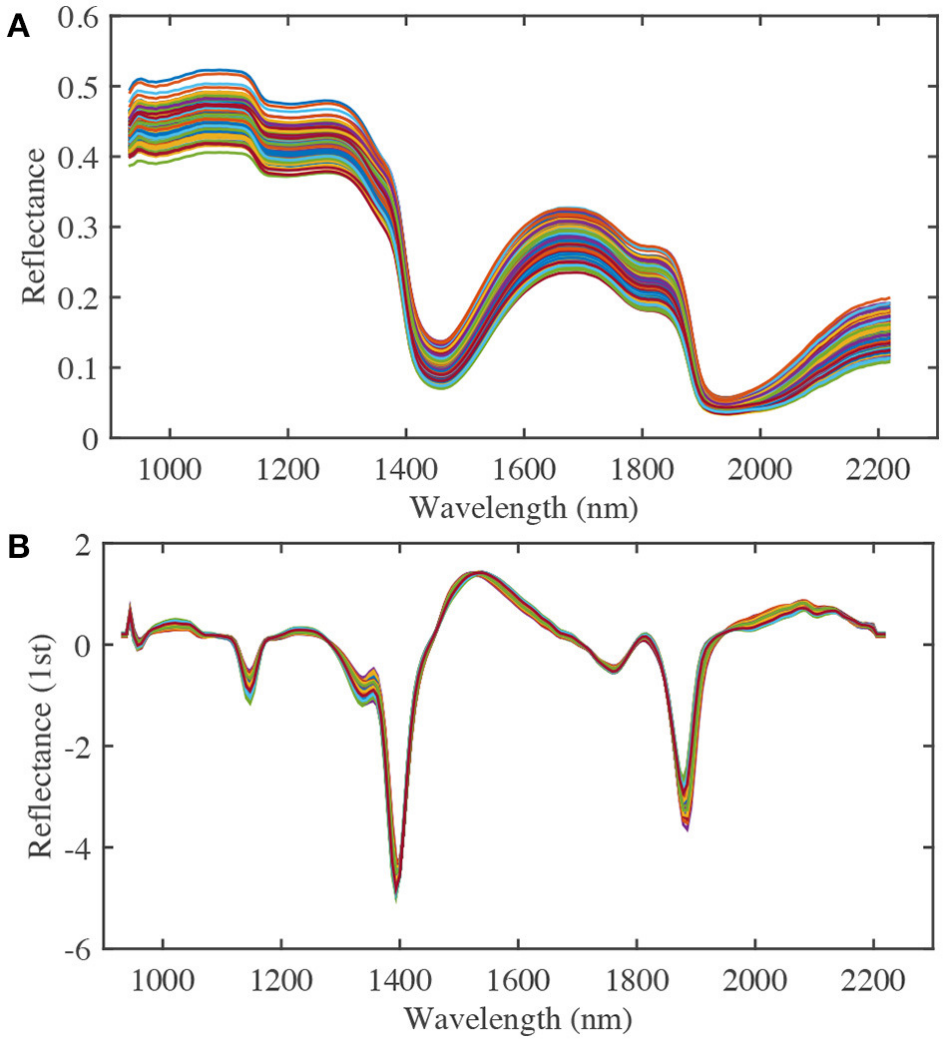
The raw **(A)** and first derivative **(B)** NIR spectra of spinach at various nitrate contents (a total of 210 samples from three nitrate fertilizer levels, each line represents a single sample).

To improve the nitrate content calibration model, different effective spectral wavelength ranges, pre-treatment methods, and numbers of PLS factors were considered. A spectral range from 932.1 to 2,217.8 nm was used in the analysis (Figure 4A). Pre-treatment of the raw spectra was required to remove the effects of interference, such as physical light scattering and baseline noise effects, and to enhance their chemical information content. Therefore, the first-derivative NIR spectra (gap-segment method: gap = 1, segment = 2; Dehghani et al., 2010) and standard normal variate (SNV) pre-treatment methods were used to construct PLS calibration models of nitrate content (Figure 4B). Finally, leave-one-out cross-validation was used to determine the optimal number of PLS factors, which showed that 16 factors had the lowest root mean square error of cross-validation (RMSECV). Determination coefficients for cross-validation (*r*^2^) and RMSECV were calculated to evaluate the different calibration models. Matlab R2014b (The MathWorks Inc., Natick, MA, USA) was used for image processing and data analysis.

### Effect of storage conditions on nitrate content and distribution

The intact leaves of similar initial state were selected and stored under four different temperature conditions (10, 20, 30, and 40°C) in a saturated humidity environment to retain a stable water content. Eight leaf samples were weighed and measured employing the NIR hyperspectral imaging every 24 h in various storage conditions, and the average values were used for analysis.

The relative water content (*RWC*) of the samples over a 4-day storage period was determined by weighing as previously described (Hunt and Rock, 1989). *RWC* was calculated according to the following equation:

(3) $$RWC=\frac{\left(FW-DW\right)}{\left(PW-DW\right)}$$

where *FW* is the fresh weight of sample (the weight of a leaf during imaging measurement), *DW* is the dried leaf weight, *PW* is the initial fresh weight of sample.

The nitrate content of the samples was then calculated from the mapping result by NIR hyperspectral imaging. The relative nitrate content (*RNC*) of the samples over a 4-day storage period was calculated according to the following equation:

(4) $$RNC=\frac{NC}{PNC}$$

where *NC* is the nitrate content of leaf a leaf during imaging measurement, *PNC* is the initial nitrate content of sample.

## Results and discussion

**Figure 5:**
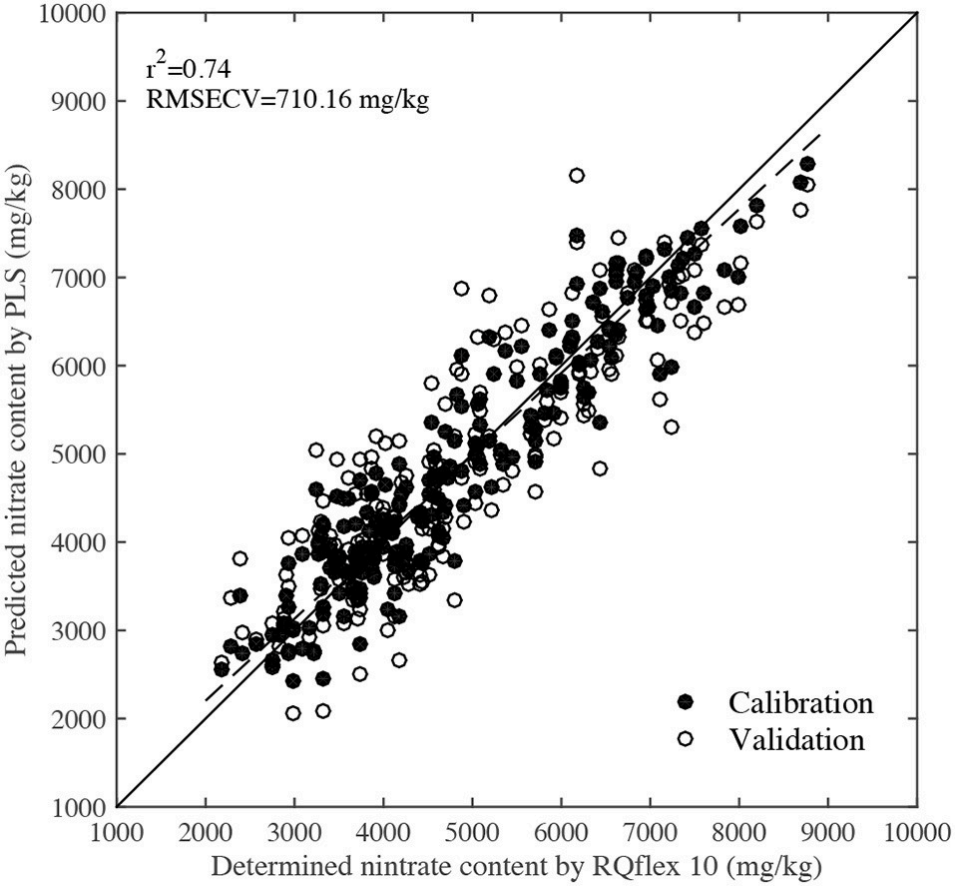
Scatter plot of predicted nitrate contents by NIR technique and determined values by RQflex 10.

Figure 5 shows the PLS calibration and validation results. The *r*^2^ and RMSECV values were 0.74 and 710.16 mg/kg, respectively, indicating that there was a high correlation between the predicted nitrate content by NIR and the determined values by RQflex 10. Reference nitrate content values of 2,000–4,000 mg/kg were usually obtained from spinach leaves grown under normal cultivation conditions, while values of 4,000–6,000 mg/kg were detected in leaves grown under high fertilization conditions. Furthermore, leaves with nitrate contents of 6,000–9,000 mg/kg were also collected from plants grown under high fertilization and dark conditions. In normal horticultural condition, the average nitrate content of spinach was around 3,000 mg/kg, but the nitrate content of veins and petioles were significantly higher than that of blades, they were more than 10,000 mg/kg. To accurately evaluate the spatial distribution of nitrate in the whole leaf including blade, vein and petiole, the calibration samples need to have a large test range. Since the PLS calibration model was established using leaves grown under a range of environmental conditions, it could be used to predict the nitrate content of most samples accurately.

**Figure 6:**
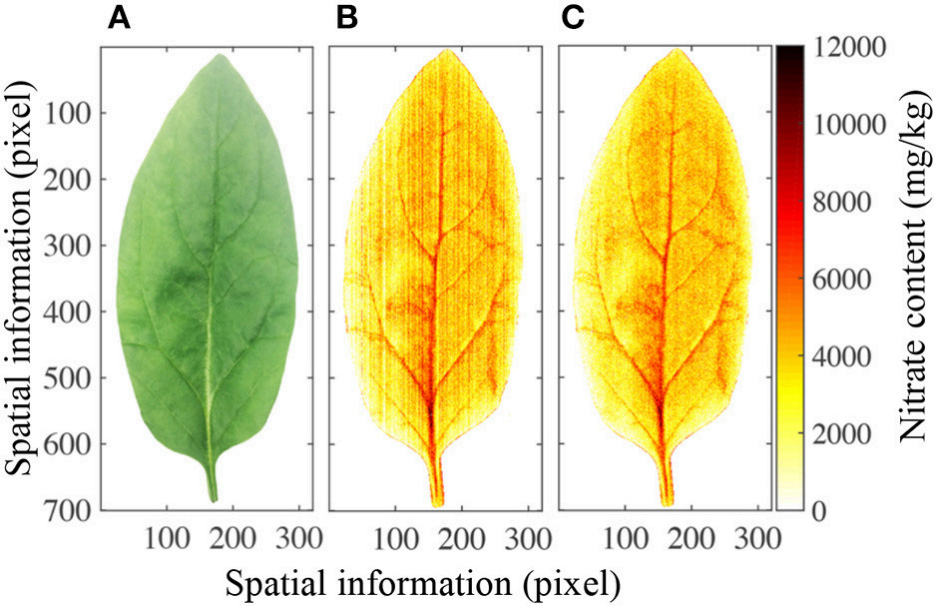
Mapping results of nitrate content in a spinach leaf. **(A)** Optical image of one spinach leaf, **(B)** nitrate content distribution in the same leaf, **(C)** nitrate content distribution in the leaf after stripe smoothing filter correction.

Figure 6A shows a photograph of a leaf as it appears to the human eye. Although the petiole, leaf vein, leaf blade, and leaf profile can be easily distinguished, the nitrate content in the leaf cannot. However, the PLS calibration model allowed the nitrate distribution to be mapped in two-dimensional space, as shown in Figure 6B, and a stripe smoothing filter could then be used to reduce the stripe spectral noise (Figure 6C). Different parts of the leaf contained significantly different nitrate contents. It has previously been shown that the nitrate contents in the petiole and leaf vein are higher than in the leaf blade, and that those in the center of the leaf are higher than at the edge (Muramoto, 1999). Furthermore, nitrate-enriched spinach plants have been shown to have 3,000–4,000 mg/kg nitrate in their leaf blades (Anjana and Iqbal, 2007; Ekart et al., 2013). All of these features can be clearly observed in the mapping image.

**Figure 7:**
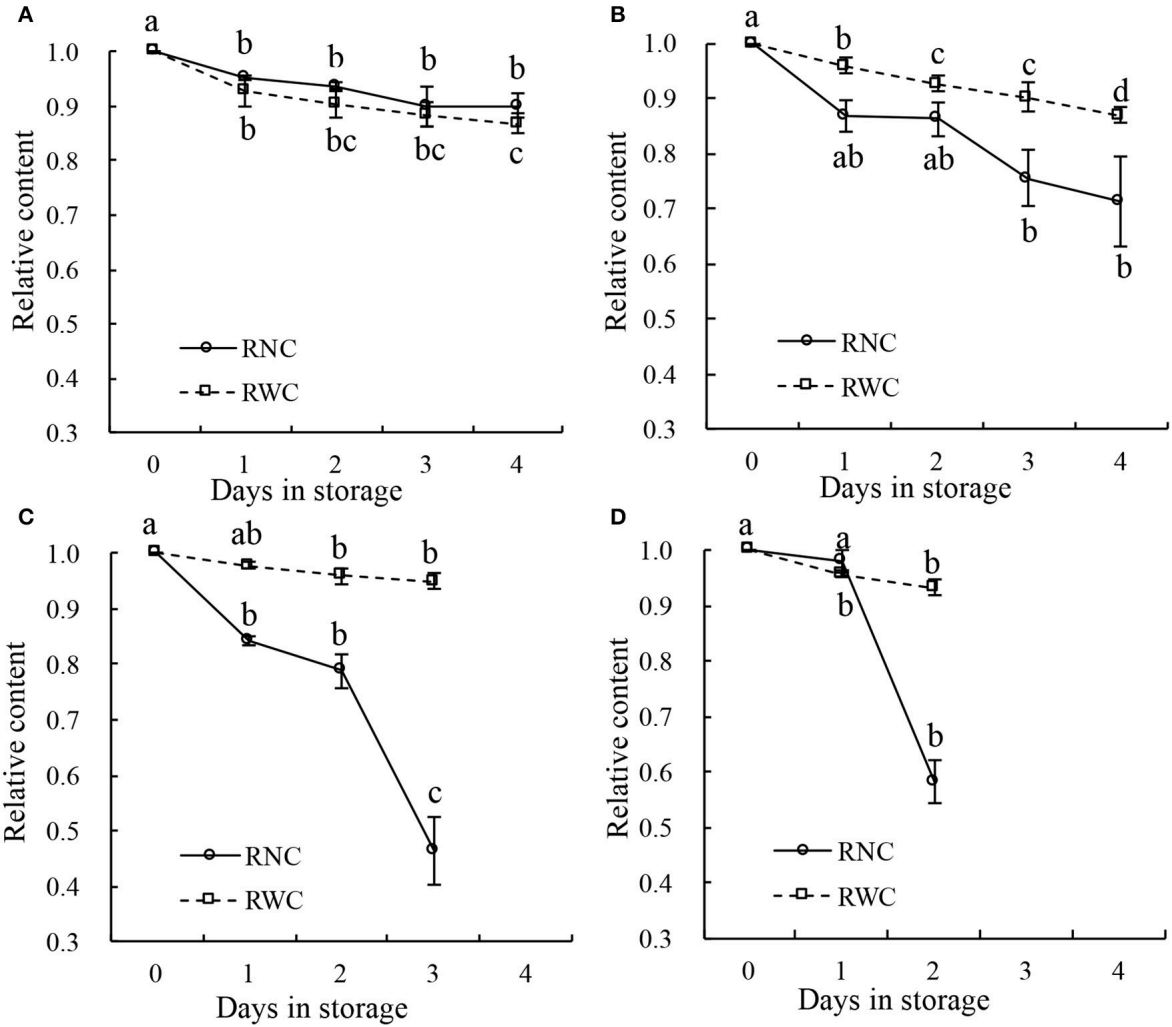
Relative water content (RWC) and relative nitrate content (RNC) of leaf samples stored for 4 days at temperature of 10°C **(A)**, 20°C **(B)**, 30°C **(C)**, and 40°C **(D)**. Lower case letters on the error bars present statistical difference at a *P* ≤ 0.05 level (by Duncan's multiple range test). Values are means ± se.

Some leaf samples were also stored under four different temperature conditions (10°, 20°, 30°, and 40°C), and the distributions of nitrate in their leaves were analyzed using NIR hyperspectral imaging. During storage at 10°C (Figure 7A), both the RWC and the RNC decreased progressively from day 1 to day 4 by 10% (such that the 1st- and the 4th-day measurements differed at *p* < 0.05). Under 20°C, both the RWC and the RNC decreased progressively from day 1 to day 4 (Figure 7B), RWC by 10%, RNC by about 20%. With the increase in storage temperature, RWC declined only by <10% till day 3 at 30°C (*p* > 0.05; Figure 7C), or till day 2 at 40°C (*p* > 0.05; Figure 7D). In contrast, RNC declined sharply by about 50% till day 3 at 30°C (*p* > 0.05; Figure 7C), or by 40% till day 2 at 40°C (*p* > 0.05; Figure 7D). Thus, while RWC declined only very gradually during the storage and its rate of decline was not affected by the storage temperature, the RNC declined with increasing speed as the storage temperature rose. These findings indicate that the differences in nitrate content distribution shown by the imaging technique were not caused by differences in water content.

**Figure 8:**
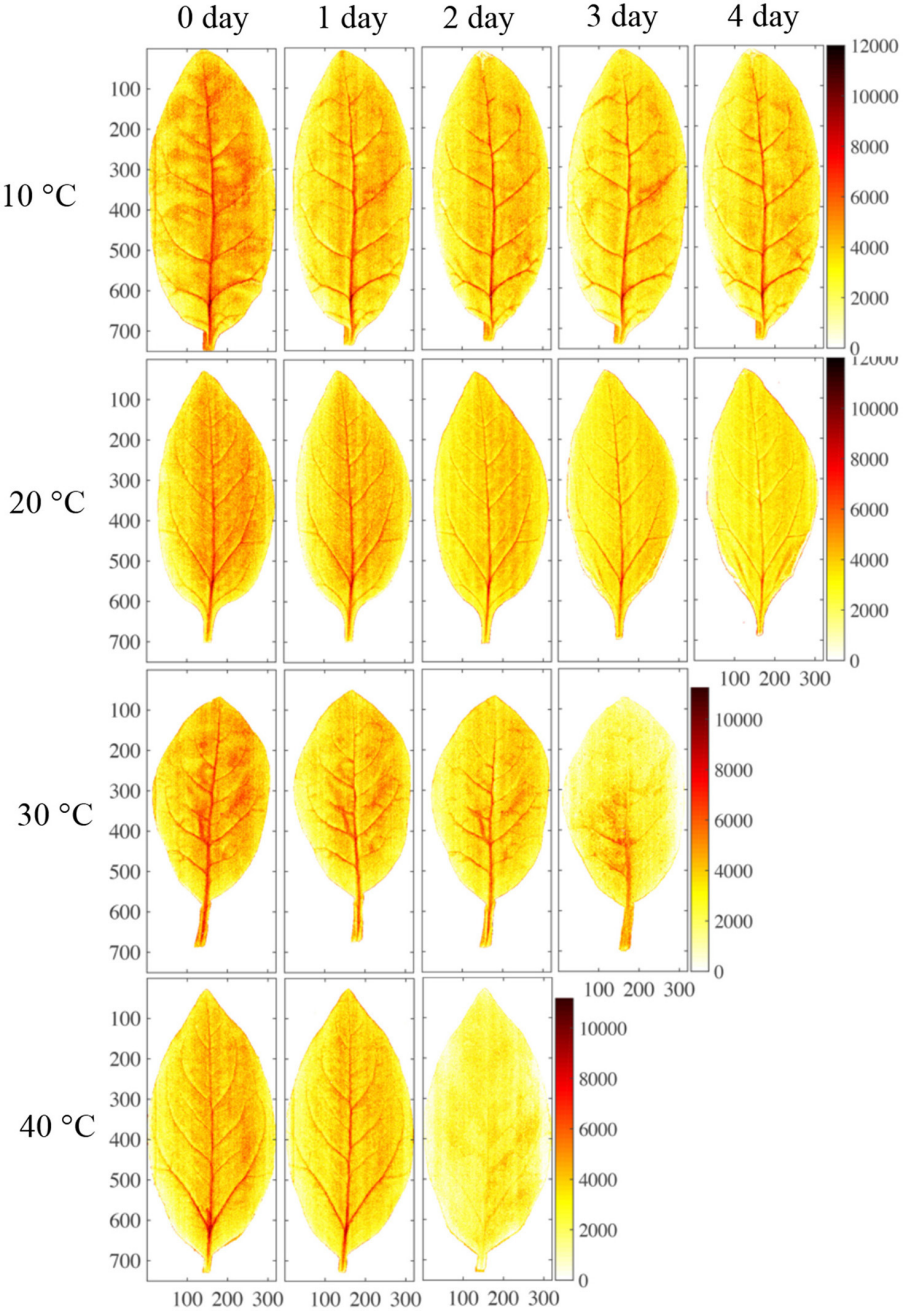
Mapping results of nitrate content in four different storage conditions.

The effects of storage temperature on the nitrate content distribution are shown in Figure 8. When stored at 10°C, the nitrate content in the sample was almost unaffected, remaining at a high level. This indicated that nitrate was not reduced to nitrite during the 4-day refrigeration period (Chung et al., 2004). Yaneva et al. (1996) also found that a cold temperature could strongly reduce the activity of nitrate reductase in the leaves of green vegetables by disturbing its internal electron transport mechanism. By contrast, nitrate content gradually decreased over the 4-day storage period at 20°C and decreased dramatically at 30° and 40°C. Nitrate content has previously been found to be reduced via microbiological reduction and nitrate reductase during storage at ambient temperature (Phillips, 1968; Yaneva et al., 1996). Therefore, the differences in nitrate content that were detected between different storage temperatures by the PLS calibration model are consistent with the findings of other studies.

## Conclusions

In this study, NIR hyperspectral imaging coupled with PLS-based analysis was found to be very effective for detecting the *in-vivo* distribution of nitrate in spinach leaves. Images were collected by the hyperspectral imaging device at different wavelengths and the resulting NIR spectroscopic data were converted into nitrate content by PLS analysis, allowing the nitrate content distribution in the leaf to be mapped in two-dimensional space. The PLS calibration model was able to predict nitrate content values accurately, both spatially within different parts of the leaf (petiole, leaf vein, and leaf blade) and between samples stored under four different temperature conditions. Thus, this method allows the rapid and non-destructive detection of nitrate content and can also be used as a tool to study the dynamic transformation of nitrate in plants.

It should be noted, however, that the estimation accuracy of the hyperspectral imaging system for detecting material components will be affected by the uneven surface of plant leaves. Therefore, to ensure that this system can detect the levels of nutrients in plant leaves with an appropriate level of accuracy, an alternative light source and advanced image processing algorithms will be required. In addition, the nitrate content prediction model constructed in this study is suitable for determining nitrate content in fresh spinach leaves as its accuracy would be degraded by leaves deterioration and water content changes.

## Author contributions

ST, H-YY, and TI designed the study; H-YY performed the laboratory experiments; H-YY and TM performed the data collection, statistical analysis and figure mapping; H-YY wrote the manuscript; ST and TI provided scientific expertise.

### Conflict of interest statement

The authors declare that the research was conducted in the absence of any commercial or financial relationships that could be construed as a potential conflict of interest.
