# Supplementary figures and images for: High-Resolution and Non-destructive Evaluation of the Spatial Distribution of Nitrate and Its Dynamics in Spinach (Spinacia oleracea L.) Leaves by Near-Infrared Hyperspectral Imaging
Source: Front Plant Sci. 2017 Nov 9;8:1937. doi: 10.3389/fpls.2017.01937 (PMC5684186; doi:10.3389/fpls.2017.01937)

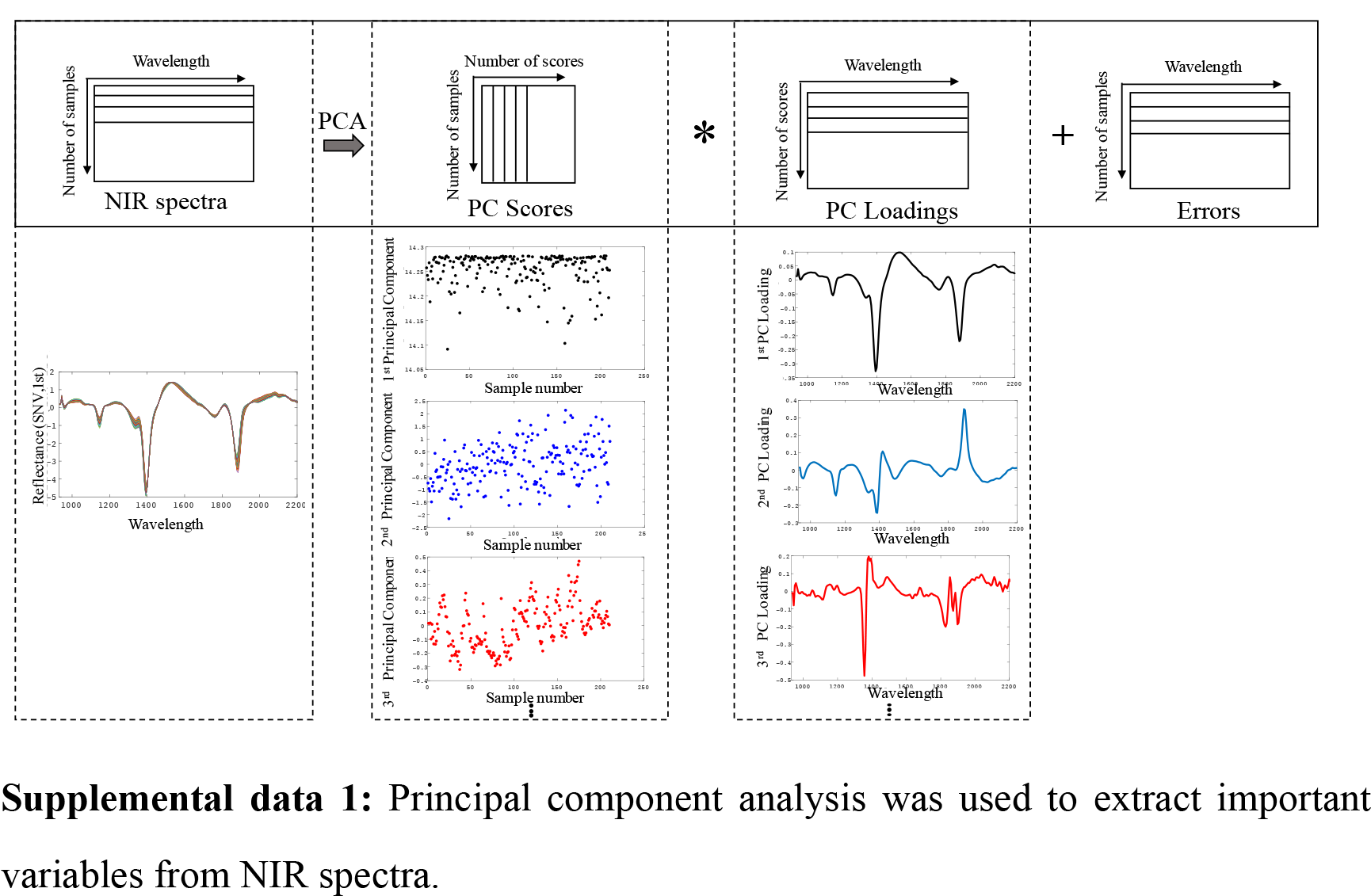

Supplement: Supplementary file 1 [file Image1.TIF]

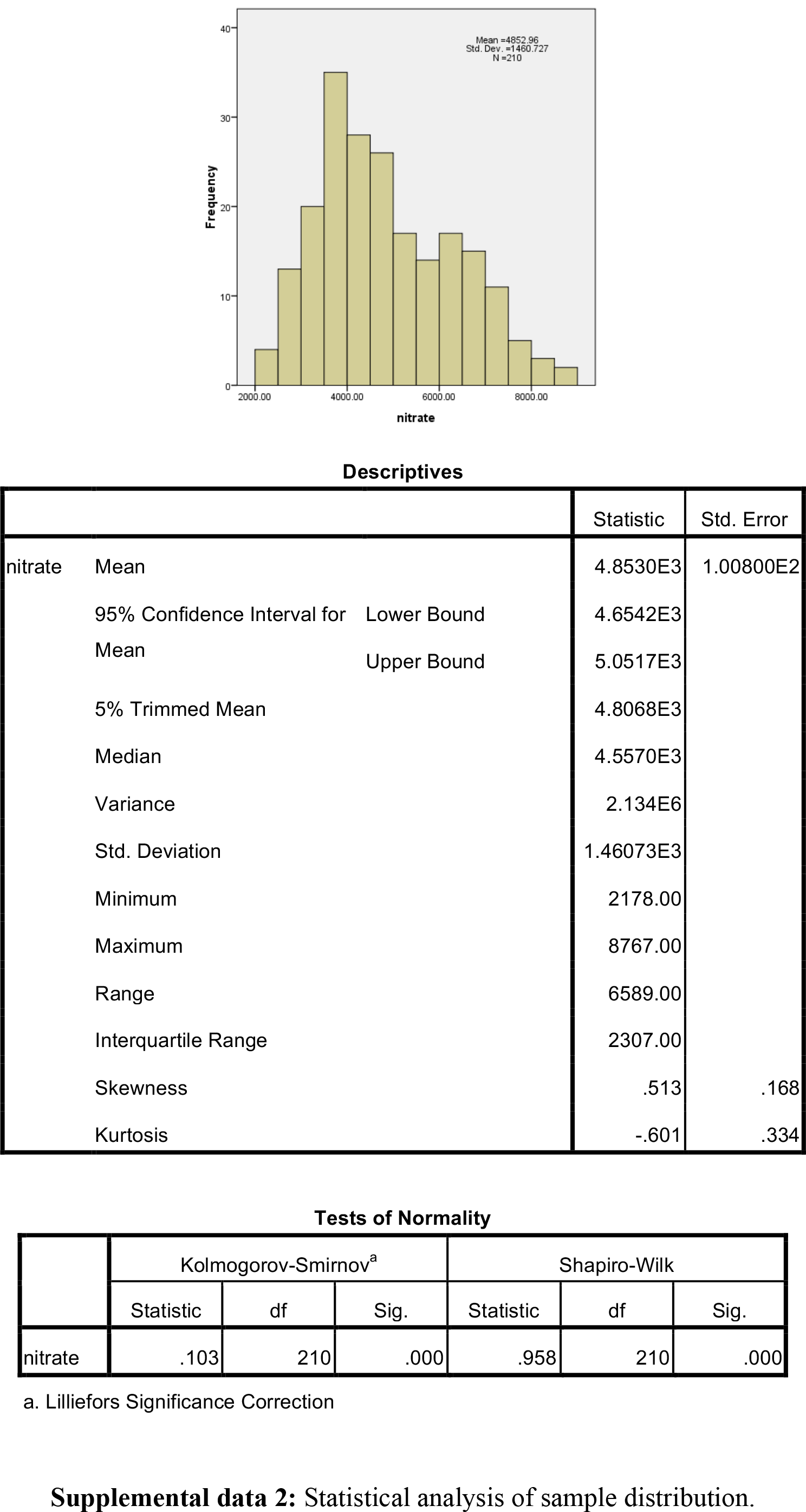

Supplement: Supplementary file 2 [file Image2.TIF]
